# Supplementary figures and images for: E3 Ubiquitin Ligase TRIP12 Controls Exit from Mitosis via Positive Regulation of MCL-1 in Response to Taxol
Source: Cancers (Basel). 2023 Jan 13;15(2):505. doi: 10.3390/cancers15020505 (PMC9856375; doi:10.3390/cancers15020505)

Supplementary Figure S1

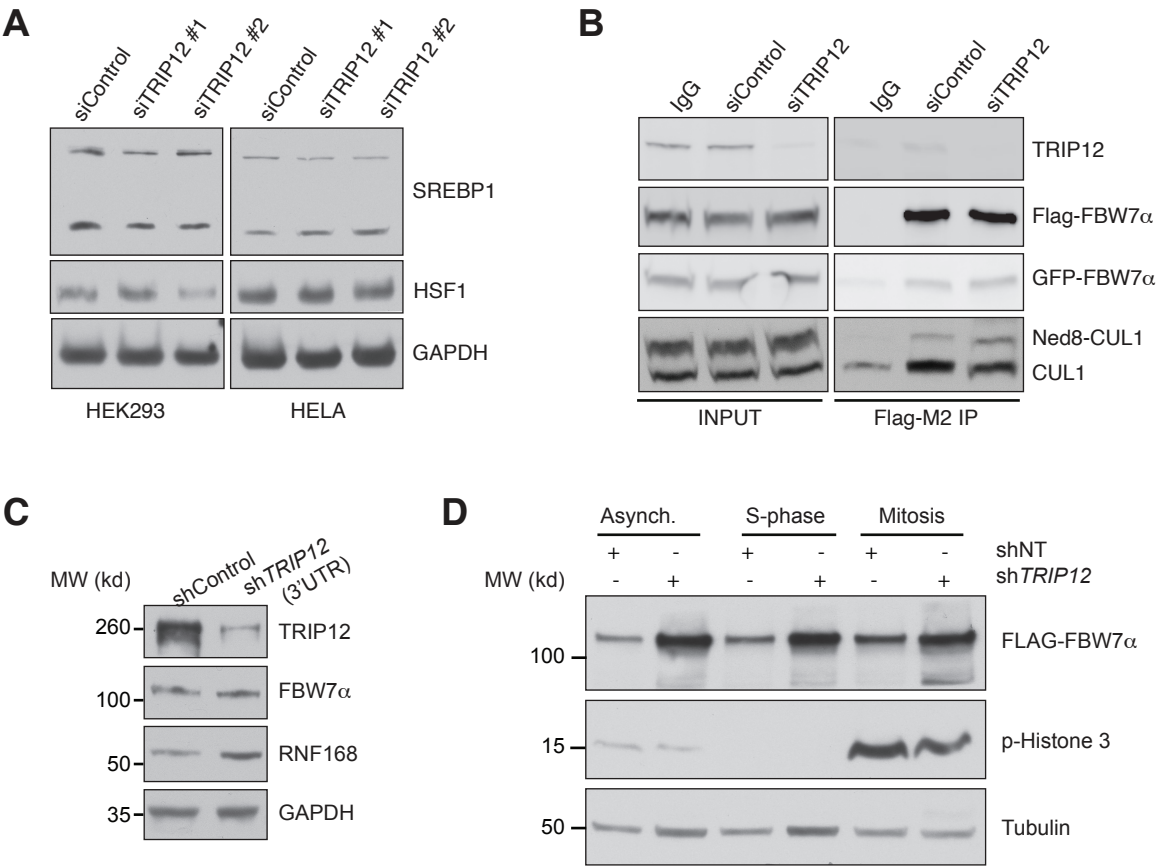

Supplementary Figure S2

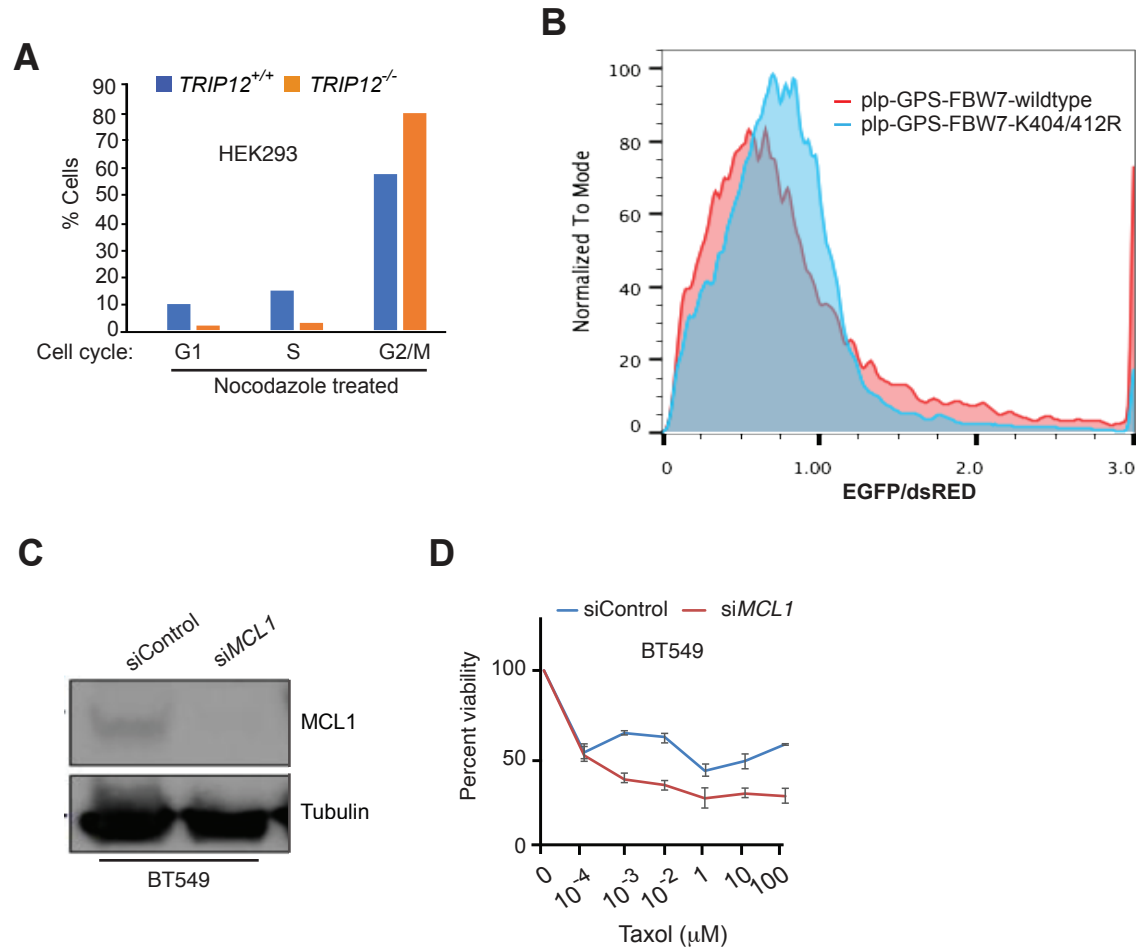

Supplement: Supplementary file 1 [file cancers-15-00505-s001.zip › cancers-2068959-supplementary.pdf]
